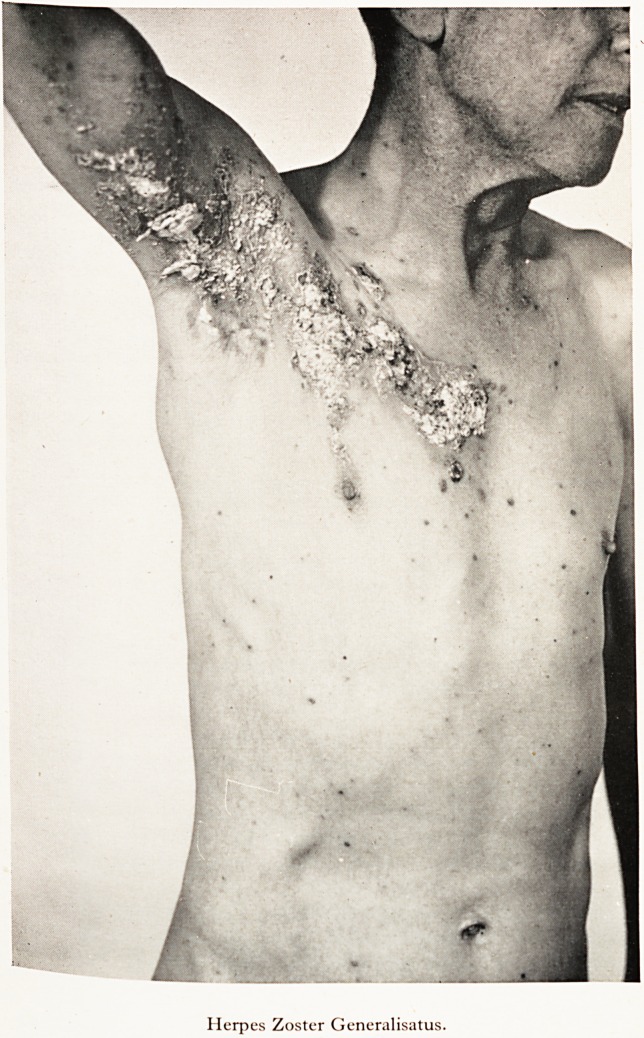# Herpes Zoster Generalisatus

**Published:** 1954

**Authors:** Mark Hewitt

**Affiliations:** Senior Registrar, Department of Dermatology, United Bristol Hospitals


### HERPES ZOSTER GENERALISATUS

BY

MARK HEWITT, M.B., M.R.C.P.

*Senior Registrar, Department of Dermatology, United Bristol Hospitals*

Herpes zoster is usually a mild illness with a characteristic painful rash and slight constitutional disturbance. The eruption is confined in the majority of cases to one or two posterior nerve root segments but a few aberrant vesicles are commonly found in the vicinity. Occasionally several nerve roots are affected and one case of universal segmental involvement has been described (Colombini, 1893).

Rarely, a generalized vesicular eruption identical with varicella complicates herpes zoster. It has been called herpes zoster generalisatus (H.Z.G.) or herpes zoster varicellosus. Four cases in chronically ill patients are described in this paper. Clinically, chicken-pox and herpes zoster are closely related and are probably caused by the same virus. Numerous cases of cross-infection have been reported; the usual sequence is that chicken-pox follows contact with zoster, the reverse being rarer. Herpes zoster generalisatus has been considered a dual infection with zoster and varicella.

Experimental support for the concept that a single virus is responsible has been provided by Brain (1933) by cross complement fixation reactions, and by Blank *et al.* (1951) who showed that in scrapings from the floor of vesicles, fixed and stained with Giemsa, the nucleoli of the giant multinuclear epithelial cells are replaced by amorphous matter and melanin granules; unique changes in zoster and varicella which cannot be demonstrated in other virus infections of skin. Brain suggests that the virus has neurotropic and dermatotropic properties which may be modified by unknown factors.

Physical weakness with a lowered resistance to infection may act as a modifying factor—it is a prominent feature in each of these cases in whom a severe attack of herpes zoster was complicated by herpes zoster generalisatus.

### CASE I

*Rheumatoid Arthritis. Chronic Bronchitis. Prostatic Obstruction. H.Z.G.*

A bus-driver, aged fifty-nine, was referred by Dr. P. S. Sinclair on account of severe stabbing pain in the right upper quadrant of his back followed three days later by a profuse haemorrhagic vesicular eruption in the second right thoracic nerve segment. Many of the vesicles became confluent and necrotic [*Plates XI and XII*], and finally cleared with extensive scarring. Between the third and fourth day after the onset of the segmental eruption several crops of isolated vesicles appeared on the face, head and neck, trunk, legs and arms. His general condition was poor, he was prematurely aged, his lips were cyanosed and he was dyspnoeic on exertion. He had a severe dorsal kyphosis; the thoracic cage was barrel-shaped and rigid, the chest expansion was limited to half an inch, and sibilant rhonchi were audible in all areas. There had been intermittent symptoms of prostatic obstruction for two years and he now developed acute retention of urine, the blood urea rising to 68 mgm. per cent. The urine was not infected and cystoscopic examination failed to reveal herpetiform lesions in the bladder, although these have been reported (Dubois, 1926) in a case of herpes zoster of the left buttock. For nine years he had been receiving treatment for chronic rheumatoid arthritis. At the time of examination the arthritis was not active but there were severe periarticular swelling, limitation of movement and chronic deformity of the hand and of the wrist, elbow, shoulder, and knee joints. Large subcutaneous nodules were present on the extensor aspects of both forearms. Biopsy revealed areas of necrobiosis and round-cell infiltration typical of those found in chronic rheumatoid arthritis.

CASE 2

*Parapsoriasis. Acute Gastric Ulcer. H.Z.G.*

A labourer, aged sixty-eight, a patient of Dr. B. E. McConnell, had noticed symptomless scattered brown pigmented scaly erythematous patches on his chest six years earlier, which had gradually increased in size and number and had become widely distributed on his trunk, legs and arms. A diagnosis of parapsoriasis was made. For three years he had each winter complained of epigastric pain made worse by food and radiating upwards to the chest and down to the abdomen and through to the inter-scapular area. The pain, with flatulence, usually lasted for eight weeks, clearing only after rest, diet and alkalis.

While undergoing treatment for a recurrence of this dyspepsia, he developed herpes zoster and was referred to the dermatological unit by Dr. G. E. F. Sutton on 10th December, 1950.

The primary lesions involving the right first and second lumbar segments had started two days previously on the thigh, pubic area and buttock. There was a profuse eruption of vesicles arranged in clusters, many were confluent and presented a linear distribution. Slightly less extensive vesiculation involving a similar area had developed in the left first and second lumbar segments. Large numbers of isolated scattered vesicles were present over the remainder of the trunk, the limbs, and the head and neck—those nearest to the primary lesions being most profuse. Fourteen days after its onset the rash had cleared.

His general condition was unsatisfactory; he had been ill at home with dyspepsia seven weeks prior to admission and had lost one stone in weight. The haemoglobin was 13·3 grams per cent. A barium meal revealed a large ulcer crater high up on the lesser curvature of the stomach. On 30th December there was severe melaena with shock and collapse, and on 4th January, 1951, the red-cell count had fallen to 1,600,000 per c.mm. and the haemoglobin to 3·7 grams per cent. Thereafter he made an uninterrupted recovery.

A fractional test meal revealed a histamine fast achlorhydria but a further barium meal and gastroscopic examination excluded a neoplasm of the stomach. The H.Z.G. had supervened in a severely ill patient with a chronic gastric ulcer. The parapsoriasis may or may not have influenced the extension of the herpes zoster.

CASE 3

*Hodgkin's Disease. Gastric Ulcers. H.Z.G.*

A housewife, aged thirty-three, a patient of Dr. B. L. Hodge, noticed a lump above her left clavicle in August, 1947, and a biopsy revealed Hodgkin's disease. Until her death in February, 1951, she was admitted to hospital on eight occasions for deep X-ray therapy to metastatic deposits in the neck, axillae, chest, supra and infra clavicular areas, sacral region, groins, the dorsal spine and sternal area. In April, 1950, she complained of pain in her back, and sclerotic metastases were detected radiologically in the 3rd, 5th, 6th, 7th and 8th thoracic vertebrae. These bones were irradiated in May, 1950, by which time she was emaciated and anaemic, and her general condition was deteriorating.

On 18th October, 1950, shortly after readmission she developed herpes zoster of the eighth left thoracic segment with severe pain and pruritus, a general constitutional disturbance and a raised temperature. On 23rd October a fresh crop of vesicles appeared in the left axilla and on the following day large numbers of vesicles developed on other areas of the chest, trunk, arms and legs. All the lesions had faded by 10th November, 1950.

A month later ascites began to complicate existing hepatic and splenic enlargement and there were irregular palpable masses in the epigastrium and right hypochondrium.

PLATE XI

Herpes Zoster Generalisatus.

PLATE XII

Herpes Zoster Generalisatus.

She began to pass large quantities of blood per rectum, and died on 24th February, 1951. A post-mortem examination revealed gross fibrous enlargement of the para-aortic glands and large masses of fibrous tissue in the portal fissure compressing the portal vein. The liver weighed 2,270 grams, had a pitted surface, was unusually lobulated, and contained irregular areas of growth. In the stomach there were two chronic ulcers one inch and half an inch in diameter on the middle third of the lesser curvature, two small chronic ulcers in the prepyloric region and one three-quarters of an inch in diameter on the posterior wall. In the chest the tracheobronchial glands appeared normal but were invested with large masses of fibrous tissue which in one area were constricting the left upper bronchus and producing collapse of the lung. The rest of the lungs were oedematous.

### CASE 4

*Seminoma Testis. H.Z.G. Herpes simplex.*

A labourer, aged thirty-five, noticed a painful swelling of the left testicle in June, 1950. Orchidectomy was performed on 1st August, 1950, and the diagnosis of seminoma was confirmed histologically. Three weeks later there was radiological evidence of lung metastases, and the chest and abdomen were treated with deep X-rays. The deposits decreased, but in November, 1950, he was readmitted on account of severe unproductive cough. Skiagrams revealed an increase in size of the lung metastases. His symptoms were immediately relieved by deep X-ray therapy and he was discharged home for a few days at Christmas.

On readmission for completion of treatment on the 2nd January, 1951, he was found to have herpes zoster. On that day, a painful pruritic eruption had developed on the left side of the chest in the second and third thoracic segments; there were clusters of vesicles of herpes simplex on the lower lip. On 7th January the eruption became generalized and vesicles appeared on the legs, trunk, face and neck and soft palate. On 9th January his general condition was worse, the eruption more profuse and, although older vesicles were fading, numerous fresh crops were appearing. The patient was dyspnoeic with a temperature of 102° F. which steadily rose to 105° F. on 11th January when he became comatose and died. H.Z.G. and bronchopneumonia were terminal infections.

Post-mortem examination showed that death was due to bronchopneumonia and myocardial failure. Herpes zoster scars were present over the left side of the chest, and more recent herpetic lesions were scattered on the face, back and legs. There was no superficial glandular enlargement. The liver contained a few firm nodules of secondary growth. The histological changes in the skin lesions were simply those of herpes zoster. The metastases consisted of a solid alveolar structure composed of large round or oval cells with a single large nucleus and a pale cytoplasm. There were numerous mitoses.

### DISCUSSION

Aberrant vesicles commonly accompany the zonal eruption of herpes zoster and are identical with the isolated vesicles of herpes zoster generalisatus. Tenneson (1893) described aberrant vesicles in nine out of ten cases of herpes zoster. Bluefarb and Morris (1941) found them in twenty out of thirty cases, and believed that they were commoner in elderly patients, particularly those with a severe infection.

Since Lippe (1889) first described a generalized vesicular eruption with herpes zoster numerous cases have been reported. Many patients have been elderly, others have had serious physical disabilities [Parounagian and Goodman (1923), Grindon (1939)]. Nobl (1911) and later Skeer (1936), Wile and Holman (1940) believe that herpes zoster generalisatus is a specific complication of chronic leukaemia, and indeed many patients with chronic lymphatic leukaemia who later developed herpes zoster generalisatus had previously received deep X-ray therapy. Ellis and Stoll (1949) consider that the effects of this treatment predispose to herpes zoster in irradiated posterior nerve roots.

Two of the present cases had been irradiated over spinal root areas whic shortly afterwards became the site of severe herpes zoster. Grave physic disabilities are a common factor in the cases described, indeed in one broncho pneumonia and herpes zoster generalisatus were terminal. The effects of age two and debility in all four may have played a major role in the general spread the zonal lesions. None of these patients had been exposed to herpes zoster varicella and no contacts were infected.

If, as seems likely, herpes zoster is due to a neurotropic factor and the general ized eruption is due to a dermotropic factor of the same virus, the clinical featur of these cases suggest that age, debilitating illness, and X-rays may in some wa modify the activity of the virus.

### SUMMARY

Four patients with herpes zoster generalisatus are described. Each had severe initial infection with zoster, and other serious physical disabilities m have been responsible for spread of the eruption.

I am indebted to Drs. S. Curwen and R. P. Warin for permission to report these cases to Dr. A. L. Taylor for details of the post-mortem findings, and to the Photograph Department of the United Bristol Hospitals.

### REFERENCES

Blank, H., Burgoon, C. F., Baldridge, G. D., McCarthy, P. L., Urbach, F. (195[illegible] *J. Amer. med. Assn.*, **146**, 1410.

Bluefarb, S. M., and Morris, G. E. (1941). *Arch. Derm. Syph.*, **43**, 385.

Brain, R. T. (1933). *Brit. J. exp. Path.*, **14**, 67.

Colombini (1893). " Caso singolarissimo di Herpes zoster universale." Siena. Quote by Blaschko, A. (1902), " Herpes ", in Mracek, F. (ed.), *Handbuch der Hautkrankheite* Vol. I, pp. 677–722. Wien, 1902.

Dubois, F. E. (1926). *J. Urol.*, **15**, 583.

Ellis, F., and Stoll, B. A. (1949). *Brit med. J.*, **2**, 1323.

Grindon, J. (1939). *Arch. Derm. Syph.*, **39**, 865.

Lippe (1889). Quoted by Schonfeld, W. (1928), " Zoster und Herpes Simplex ", i Jadassohn, J. (ed.), *Handbuch der Haut- und Geshlechtskrankheiten*, Bd. 7, Teil 1, p. Berlin.

Nobl, G. (1911). " Zur Kermtnis des Herpes zoster generalisatus ", *Wien Klin. Wschr* **24**, 14.

Parounagian, M. B., and Goodman, H. (1923). *Arch. Derm. Syph.*, **7**, 439.

Skeer, J. (1936). *Arch. Derm. Syph.*, **34**, 809.

Tenneson, H. (1893). *Traite clinique de dermatologie*, 116. Paris.

Wile, U. J., and Holman, H. H. (1940). *Arch. Derm. Syph.*, **42**, 587.

## Figures and Tables

**Figure f1:**
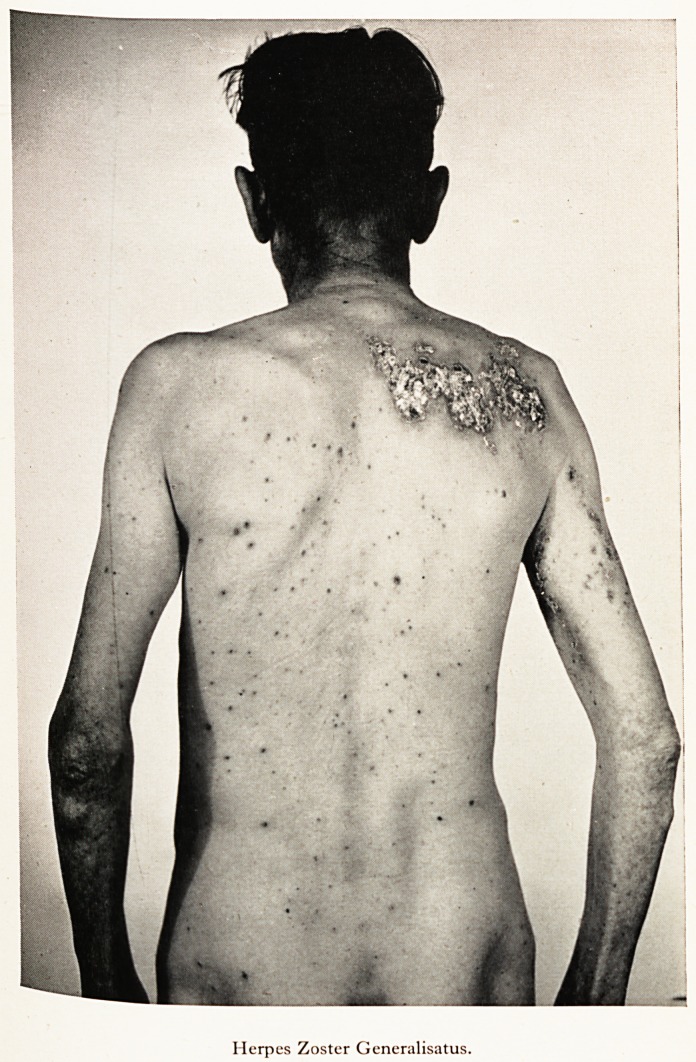


**Figure f2:**